# Glucocorticoid induced loss of oestrogen receptor alpha gene methylation and restoration of sensitivity to fulvestrant in triple negative breast cancer

**DOI:** 10.1016/j.gene.2022.147022

**Published:** 2022-11-05

**Authors:** Haya Intabli, Julia M. Gee, Steffi Oesterreich, Mark S. Yeoman, Marcus C. Allen, Amal Qattan, Melanie S. Flint

**Affiliations:** a Centre for Stress and Age-Related Disease, School of Pharmacy and Biomolecular Science, University of Brighton, Brighton BN2 4GJ, UK; b Breast Cancer Molecular Pharmacology Group, School of Pharmacy and Pharmaceutical Sciences, Cardiff University, Wales CF10 3NB, UK; c Department of Pharmacology and Chemical Biology, Women’s Cancer Research Center, UPMC Hillman Cancer Center and Magee Womens Research Institute, Pittsburgh, PA 15232, USA; d Translational Cancer Research Section, Department of Molecular Oncology, King Faisal Specialist Hospital and Research Centre, Riyadh 11211, Saudi Arabia; e UPMC Hillman Cancer Center, Pittsburgh, PA, USA; f Magee-Women’s Research Institute and Women’s Cancer Research Center, Pittsburgh, PA, USA

**Keywords:** Stress, Breast cancer, Epigenetics, Methylation

## Abstract

The response to psychological stress can differ depending on the type and duration of the stressor. Acute stress can facilitate a “fight or flight response” and aid survival, whereas chronic long-term stress with the persistent release of stress hormones such as cortisol has been shown to be detrimental to health. We are now beginning to understand how this stress hormone response impacts important processes such as DNA repair and cell proliferation processes in breast cancer. However, it is not known what epigenetic changes stress hormones induce in breast cancer. Epigenetic mechanisms include modification of DNA and histones within chromatin that may be involved in governing the transcriptional processes in cancer cells in response to changes by endogenous stress hormones. The contribution of endogenous acute or long-term exposure of glucocorticoid stress hormones, and exogenous glucocorticoids to methylation patterns in breast cancer tissues with different aetiologies remains to be evaluated. *In vitro* and *in vivo* models were developed to investigate the epigenetic modifications and their contribution to breast cancer progression and aetiology. A panel of triple negative breast cancer cell lines were treated with the glucocorticoid, cortisol which resulted in epigenetic alteration characterised by loss of methylation on promoter regions of tumour suppressor genes including *ESR1*, and loss of methylation on LINE-1 repetitive element used as a surrogate marker for global methylation. This was verified *in vivo* in MDA-MB-231 xenografts; the model verified the loss of methylation on ESR1 promoter, and subsequent increase in ESR1 expression in primary tumours in mice subjected to restraint stress. Our study highlights that DNA methylation landscape in breast cancer can be altered in response to stress and glucocorticoid treatment.

## 1. Introduction

Breast cancer is molecularly classified based on three main biomarkers: oestrogen receptor (ER), progesterone receptor (PR), and human epidermal growth factor receptor 2 (HER2) (Lee et al., 2019). Approximately 75% of breast tumours are ER positive, known as Luminal A, and Luminal B breast cancer (Sharma et al., 2018; Miah et al., 2019). Breast cancers that lack ER, PR, and HER2 are known as Triple Negative Breast Cancer (TNBC), and they account for 15% to 20% of breast cancer tumours (Lee et al., 2019; Garrido-Castro et al., 2019). This molecular characterisation is crucial at diagnosis as it dictates the type of the therapy the patient receives and plays a role in predicting clinical outcomes. Patients with tumours expressing ER are candidates for endocrine therapy (Billam et al., 2009; Selli et al., 2016), while patients with tumours lacking ER expression are unresponsive to endocrine therapy, as these therapies are molecularly targeted therapies that work by modulating or antagonising ER, thus inhibiting the growth of the tumour (Billam et al., 2009; Gucalp and Traina, 2011; Mast and Kuppusamy, 2018; Schiff and Osborne, 2005).

Steroid receptors including ER, PR, glucocorticoid receptor (GR), and androgen receptor (AR) have been shown to play an important role in breast cancer progression (Tonsing-Carter et al., 2019). Although the role of ER and PR in breast cancer is relatively well established, the orchestrated functional interactions of these nuclear receptors are now being recognised (Tonsing-Carter et al., 2019; Miranda et al., 2013). Increasing evidence demonstrates a role of GR in the interaction with nuclear receptors in breast cancer progression. For example, the activation of PR, AR, and GR leads to the modification of ER-mediated gene expression in ER+ breast cancer (Tonsing-Carter et al., 2019).

Oestrogen is a key modulator for the development of normal and malignant breast tissues. The crosstalk between GR and ER has also been recognised; it has been shown that the activation of GR by dexamethasone (DEX) plays a crucial role in the metabolic deactivation of oestrogens. This has great implications in breast cancer therapeutic development (Gong et al., 2008; Karmakar et al., 2013).

Breast cancer tumours lacking ERα expression are unresponsive to hormonal treatment, such as oestrogen receptor modulators and oestrogen receptor down-regulator (Miah et al., 2019). This loss of expression can be attributed to genetic and epigenetic causes (Billam et al., 2009). However, compelling evidence from the literature suggests that it’s the epigenetic changes, which are the main cause behind the ER silencing (Billam et al., 2009; Yan, 2003; Prabhu et al., 2012; Sharma et al., 2006; Ramezani et al., 2012; Yang, 2001). The loss of ERα in ER-negative breast cancer cell lines has been associated with the downregulation of *ESR1* mRNA expression, which can be a result of methylation of CpG islands on the promoter region and exon 1 of *ESR1* (Billam et al., 2009; Sharma et al., 2006). The fact that the methylation process is mainly governed by the DNMT family of enzymes (Yan, 2003) and that the maintenance methyltransferase DNMT1 plays a role in demethylation (He et al., 2017), directed studies into investigating the role of DNMT1 in ER silencing. Furthermore, *DNMT1* mRNA and protein levels were found to be elevated in ER-negative cell lines when compared to ER-positive cell lines (Yoshida, 2000; Sogon, 2007; Pinzone, 2004). The repressing transcription mechanism of ERα in breast cancer is orchestrated either solely by DNMTs, or by a combination of DNMTs and HDACs, and corepressor complex elements (Sharma et al., 2006; Yang, 2001; Zhou et al., 2007; Bicaku et al., 2008; Yang, 2000; Sharma, 2005; Kawai et al., 2003).

Data suggesting that ER is silenced epigenetically led to investigations into the possibility of re-expressing ER and restoring its functionality and hormonal sensitivity to provide a therapeutic target for ER-negative breast cancer. Different approaches have been used to facilitate the re-expression of ER; such as DNMT inhibitors, HDAC inhibitors, and a combination of both. One study demonstrated that treatment of ER-negative breast cancer cells with a demethylating agent and the DNMT1 inhibitor, 5-aza-2′deoxycytidine (5-aza-dC), led to the re-expression of ER mRNA and functional ER proteins, by specifically inhibiting DNMT1 expression (Yan, 2003; Yang, 2001; Huang et al., 2012). In addition, a combination of (5-aza-dC) and the HDAC specific inhibitor, trichostatin A (TSA), enhanced effect of ER re-expression in TNBC cells (Yang, 2001). Another study demonstrated the re-expression of ER-α by TSA in a cell line that had lost ERα expression (Kawai et al., 2003).

Although several studies have been summarised illustrating the effects of glucocorticoids (GC’s) on DNA methylation in different models, little is known about the effects of cortisol on the epigenetic modification of breast cancer. Herein, we examined the influence of cortisol on DNA methylation in cells and animal models of breast cancer. We also investigated the methylation status of *ESR1* in a panel of TNBC cell lines, along with the mRNA expression of *ESR1* in response to long-term exposure to cortisol. Further studies were conducted to test whether the activation of hypothalamic pituitary adrenal (HPA) axis in response to psychological restraint stress would have a similar effect on the methylation and expression status of ER. Using a TNBC MDA-MB-231 xenograft model we found that the expression of ESR1 significantly increases in the tumours of stressed mice. We demonstrated the decrease in *DNMT1* in response to cortisol treatment in TNBC but not *in vivo* and found changes in methylation status of key genes including loss of methylation on the promoter region of *ESR1*.

## 2. Materials and methods

### 2.1. Cell culture

MDA-MB-231, MDA-MB-157, MCF-7, Hs-578T, T47D and BT-549 TNBC cells were obtained from the American Type Culture Collection (ATCC). These cells have been previously reported to express GR (Reeder et al., 2015). MDA-MB-231, MDA-MB-157, and Hs578T cells were grown in Dulbecco’s Modified Eagle Medium Nutrient Mixture F-12 (Ham) (DMEM/F-12 in a 1:1 ratio) with 10% foetal bovine serum (Gibco). BT-549 cells were grown in Roswell Park Memorial Institute medium (RPMI-1640) with 0.023U/ml insulin and 10% foetal bovine serum (Gibco). MCF-7 and T47D cells express ER, PR, but not HER-2, and hence represent a luminal subtype of breast cancer. MCF-7 cells express higher levels of GR, while in comparison; T47D cells have low GR expression (Reeder et al., 2015). MCF-7 and T47D cells were grown in Dulbecco’s Modified Eagle Medium Nutrient Mixture F-12 (Ham) (DMEM/F-12 (1:1)) with 10% foetal bovine serum (Gibco).

### 2.2. Hormone treatment

Prior to hormone treatment, culture media was replaced with phenol-red-free media of the appropriate media type containing 10 % charcoal stripped media for at least 48 hrs. Growth media were replaced every 24 hrs with fresh cortisol diluted in media for 20 days unless stated otherwise. RU-486 was used as an antagonist for GR. RU-486 (Sigma, UK) was dissolved in absolute ethanol and diluted in water then in appropriate culture media to achieve a final concentration of 1 μM. RU-486 was added 30 min prior to cortisol treatment (Huang et al., 2012). Dexamethasone (Dex) (Sigma Aldrich, UK) was diluted in absolute ethanol and further diluted in appropriate culture media to achieve final concentrations of 1 μM, 0.5 μM, and 0.25 μM. Growth media were replaced every 24 hrs with Dex diluted in media.

### 2.3. Microculture Tetrazolium (MTT) assay

Cells were plated at a density of 3000 cells/200 μl in 96-well plates in hextuplicates and incubated for 24 hrs. Cells were then treated with either 0.1 μM Fulvestrant (Sigma Aldrich, UK), 1 μM Tamoxifen (Sigma Aldrich, UK), or/and 10 nM Estradiol. After 7 days of incubation, the culture media was replaced with 200 μl of 0.2 mg/ml MTT powder dissolved in the appropriate media of the cell line. Cells were incubated with MTT for 2 hrs at 37 °C protected from light. The MTT solution was then removed and 200 μl of dimethyl sulfoxide (DMSO) was used to dissolve the formazan crystals. The absorbance was read using a spectrophotometer at a wavelength of 495 nm. Considering that the absorbance is proportional to the number of viable cells, the viability was expressed as a percentage of the control wells in each experiment.

### 2.4. Nucleic acid extraction

RNA was extracted from cell pellets, or homogenised tissues using RNeasy mini Kit (Qiagen Cat No.74104) according to the manufacturer instructions. cDNA was synthesised from 1 μg total RNA using High-Capacity cDNA Reverse Transcription Kit using QuantiTect Reverse Transcription Kit (Qiagen Cat No. 205311) as per manufacturer’s instructions. DNA was extracted from cells and tissues using Gentra Puregene kit (Qiagen Cat No: 158767) according to manufacturer’s manual. DNA and RNA were quantified using NanoDrop 2000 spectrophotometer from NanoDrop Technologies (Thermofisher UK). The 260/280 helps assess the purity of DNA and RNA preparation; DNA was considered pure when 260/280 was ≈ 1.8 and RNA considered pure when 260/280 ≈2.0.

### 2.5. Gene expression analysis

To assess the change in gene expression in cells, real-time PCR was performed. SYBR green-based assay was used to study gene expression from cDNA synthesized from cells, or tissue. Rotor-Gene Q PCR instrument from Qiagen was used, master mix from Rotor-Gene SYBR Green PCR kit (Qiagen Cat No. 204074), and bioinformatically verified primers were bought from Qiagen. Primers include ESR1, DNMT1, DNMT3a, DNMT3b, and β-Actin. Primers are provided in a lyophilized mix of forward and reverse primers and are reconstituted in 1.1 ml of (Tris EDTA) buffer, pH 8.0 to obtain a 10x assay solution. Gene expression was using the ΔΔC_t_ method.

### 2.6. Profiler array

Two PCR profiler arrays were bought from QIAGEN to study the expression of multiple genes, the Human Estrogen Receptor Signalling array, and Human Epigenetic Chromatin Modification Enzymes PCR Array (Qiagen Cat No. 330231). RNA was extracted and cDNA was synthesised from 500 ng of RNA as described earlier. Samples were then mixed with 1350 μl of SYBR Green (QIAGEN), and volume was adjusted with RNase-free water to a final volume of 2700 μl of PCR master mix. An equal volume of 25 μl of PCR components mix was loaded to each well containing the primers for different genes. β-Actin was used as an endogenous control, and gene expression was quantified using the ΔΔC_t_ method. A list of genes can be found in Supplementary Table 1.

### 2.7. DNA methylation profiling using MethylScreen technology

EpiTect Methyl II PCR Array Human Tumour Suppressor Genes, Complete panel (Qiagen) was used to analyse the change in promoter methylation status of 94 different tumour suppressor genes in response to cortisol treatment. MDA-MB-231 and MCF7 cells were treated with cortisol for 20 days as described earlier. DNA was then extracted from cells using Gentra Puregene Cell kit (Qiagen) as per manufacturer’s manual. 4 μg of DNA was divided into four digestion reactions using the EpiTect Methyl II DNA restriction Kit (Qiagen) a per manufacturer’s protocol. The kit provides a methylation sensitive and a methylation dependent restriction enzyme that are designed to digest methylated cytosine and un-methylated cytosine respectively. The enzymes are incubated with the DNA over night at 37 °C along with a sample with both digestion enzymes and a sample with no enzymes for normalisation. The digested DNA was then analysed using the EpiTect Methyl II PCR array as per manufacturer’s instructions. The PCR assay was performed in Applied Biosystems ViiA 7 real time PCR system, and data were analysed using an integrated Excel-based template provided by the EpiTect Methyl II PCR array system. This MethylScreenTM technology system automatically calculates the percentage of DNA that is methylated or unmethylated using the raw Ct values uploaded to the system available at: https://www.sabiosciences.com/dna_methylation_data_analysis.php Results were calculated using ΔΔCt method. List of genes can be found in Supplementary Table 2.

### 2.8. LINE-1 analysis

DNA was extracted using Gentra Puregene Cell kit (Qiagen) according to manufacturer’s protocol. 1 μg of genomic DNA was used in LINE-1 (Long interspersed nuclear elements) kit; an ELISA- based assay (Active motif), for the detection and quantification of global DNA methylation as per manufacturer’s instructions. LINE-1 are transposable elements that comprise of approximately 17–18% of the human genome; hence quantification of LINE-1 methylation levels could serve as a surrogate of global DNA methylation.

### 2.9. Western blots

Cells treated with hydrocortisone and their controls were plated at a density of 5×10^5^ cells per well. Cells were then washed thoroughly with cold PBS and lysed with RIPA Buffer (Sigma Aldrich, R0278) at 4 °C on an agitating thermomixer (Eppendorf), for 1 hr. The lysate was then centrifuged at 13,000*g* for 20 min at 4 °C. The supernatant was then collected to measure total proteins using Bradford Assay (Sigma Aldrich).

A total of 30 μg of protein was loaded into precast gel (4–20%) and then transferred onto polyvinylidene fluoride (PVDF) membranes. Membranes were then blocked using 5 % skimmed milk for 1 hr, and then incubated with DNMT1, DNMT3a, β-actin antibodies (1:1000) (Cell Signalling), and ERα antibody 1:500 (Santa Cruz) in 5 % skimmed milk overnight on a shaker at 4 °C. Membranes were then washed with TBST (Sigma Aldrich) 3 times 10 min each, then incubated with appropriate secondary antibody (Ani-rabbit/ mouse 1:2000, Cell Signalling) and (Anti mouse/ goat 1:2000, Promega) in TBST for 1 h on a shaker at room temperature. Membranes were then washed with TBST and rinsed with PBS. Signals were detected with Amersham ECL reagents western blotting detection reagent kit (GE Healthcare) and bands were imaged on an Image Quant LAS 400 imager (GE Healthcare). The optical densities of the bands were analysed using ImageJ software.

### 2.10. Xenografts

Female nude BALB/c mice were injected with 5×10^6^ MDA-MB-231 cells in 0.2 ml of PBS into the fourth left mammary fat pad. Mice were handled daily for 2 weeks to acclimatise to the investigator. The mice were weighed once every week and tumour volumes were measured every other day using digital callipers. The tumours took 8–10 weeks to establish tumour volumes of 50 mm^3^ – 75 mm^3^. When tumours reached ≈ 50 mm^3^, tumour-bearing xenografts were randomised into stressed (RS) (n = 4) and non-stressed (NS) (n = 4) groups. This is a well-established stress model that has shown previously to induce cortisol in this *in vivo* model system (Mujoo et al., 2014). Stressed mice were placed individually in ventilated 50 ml conical tubes for 2 hrs daily at the same time from 10 am to 12 am. Mice can turn prone to supine but not head to tail. Mice were sacrificed when any parameter of the volume reached a maximum of 12 mm^3^. All primary tumours were harvested at necropsy.

### 2.11. Immunohistochemistry

Tumours of xenografts were paraffin-embedded, then cut into 5 μm-thick transverse sections for immunohistochemical assessment. Staining was performed at the University Hospitals Sussex NHS Foundation Trust using EnVision FLEX, High pH (Dako Omnis) kit for ER staining (Agilent GV 800) as per manufacturer’s instructions. Sections were first deparaffinised in serial ethanol, and then submerged in high pH solution for 30 min to perform antigen retrieval. Samples were then incubated with primary antibody against ER (Dako M3643) at a dilution (1:40) for 20 min. Samples were further incubated with hydrogen peroxide blocking agent for 3 min followed by incubation with a rabbit linker for 10 min to amplify signals. Samples were then incubated with horseradish peroxidase polymer for 20 min. Samples were finally stained with a visualising chromogen for 5 min and counterstained with haematoxylin for 3 min. Samples were then obtained and scored blindly from 0 to 5 where 0 = no cells are stained, 1= <1% of cells are stained, 2 = 1–10% of cells are stained, 3 = 11–33% of cells are stained, 4 = 34–66% of cells are stained, and 5 = 67–100% of cells are stained (Ilić et al., 2019).

### 2.12. Statistical analysis

For continuous data assuming normal variance, one-way analysis of variance was used with Bonferroni multiple comparisons test between groups. For discrete data, the Mann-Whitney test was used. Statistical significance was determined when the *p* value was < 0.05. All the results are representative of the mean of three independent experiments (*n* = 3), each with three technical replicates ± SEM unless otherwise stated. qRT-PCR data were analysed as relative quantification normalised against control. Results were presented as mean ± SEM, and one sample *t*-test was used to compare the mean significance to a hypothetical value of 0 (control). GraphPad Prism was used for all statistical analysis.

## 3. Results

### 3.1. Cortisol induced specific promoter region methylation alterations and LINE-1 methylation changes in breast cancer cell lines

**Fig. 1.**
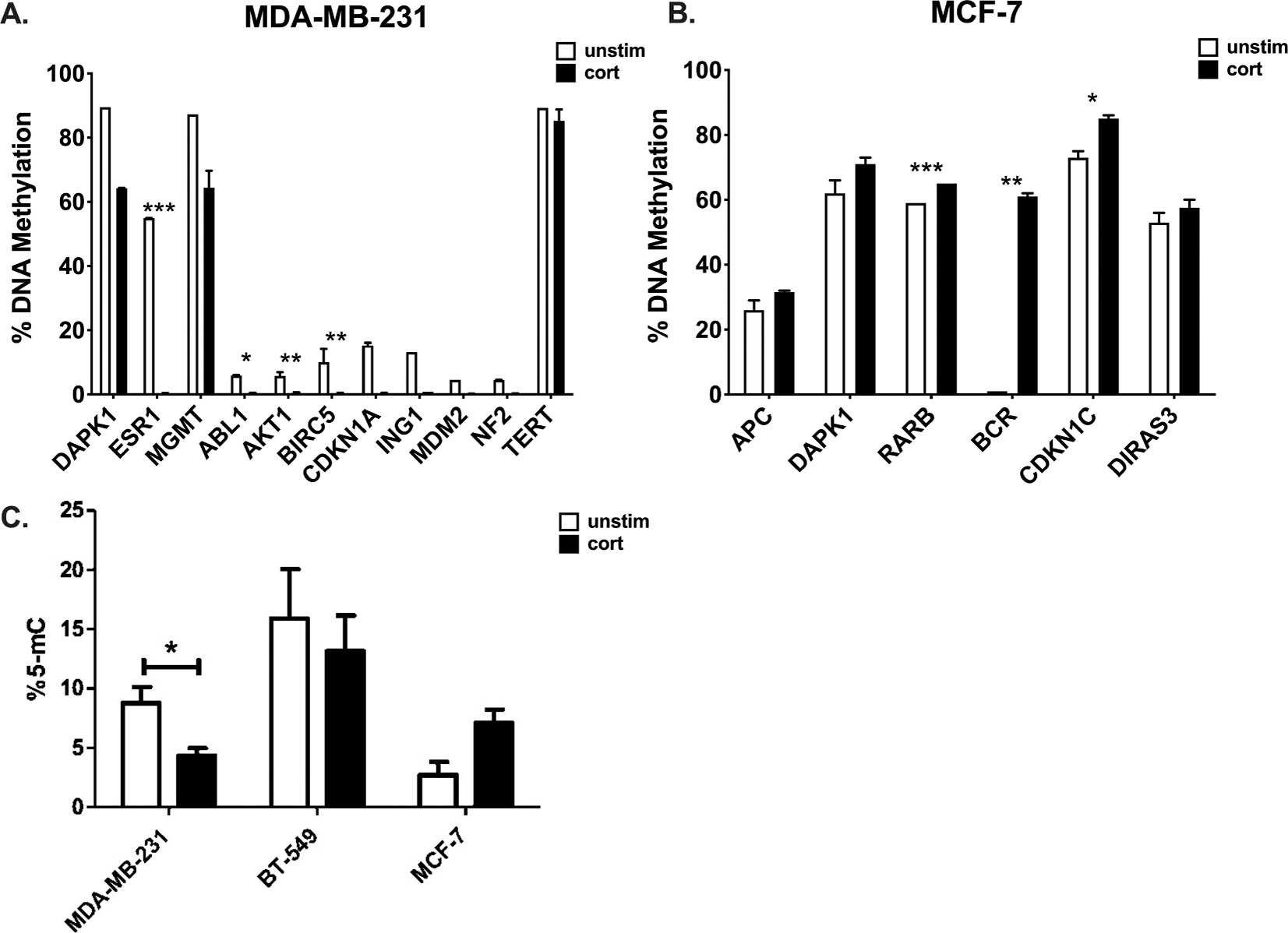
Specific promoter region methylation changes and changes of LINE-1 methylation in response to cortisol treatment in breast cancer cells. A. qRT-PCR on Human Tumour Suppressor Array on MDA-MB-231 cells. B. qRT-PCR in MCF-7 cells. C. The percentage of methylated cytosine correlated with detectable CpG residues in LINE-1 in MDA-MB-231, BT-549, and MCF-7 cells in response to 20 days of treatment with cortisol. Mean ± SEM expressed and multiple *t*-test with Bonferroni correction was used to compare untreated (unstim) and treated (cort) samples. * Represents significant difference (* p < 0.05, ** p < 0.01, ***p < 0.001).

To investigate the epigenetic effects of cortisol exposure on breast cancer, MDA-MB-231 and MCF-7 cells were exposed daily to 5 μM cortisol for 20 days. DNA was extracted and used for qRT-PCR of EpiTect Methyl II PCR Array Human Tumour Suppressor Genes. MDA-MB-231 cells exposed to cortisol showed a decrease in methylation levels of the following tumour suppressor genes *DAPK1, ESR1, MGMT, ABL1, AKT1, BIRC5, CDKN1A, ING1, MDM2, NF2, PYCARD, and TERT* compared to untreated cells (Fig. 1B). However, significant changes were detected for *ESR1* (p < 0.001), *ABL1* (p < 0.05), *AKT1* (p < 0.01), and *BIRC5* (p < 0.01) suggesting a potential activation of these genes (Fig. 1A). MCF-7 cells exposed to cortisol, demonstrated changes in the following tumour suppressor genes: *APC, DAPK1, RARB, BCR, CDKN1C, and DIRAS3.* However, significant increases in methylation were detected in *RARB* (p < 0.001), *BCR* (p < 0.001), and *CDKN1C* (p = 0.024) implying potential silencing or down regulation of the expression of these key genes (Fig. 1B).

To investigate the effect of cortisol of global DNA methylation patterns, the change in the percentage of methylated cytosines on the repetitive element LINE-1 was assessed in MDA-MB-231, BT-549 and MCF-7 cells exposed to cortisol. Since LINE-1 comprises for a bulk of human genome and around third of methylation incidents appears to be on repetitive elements, LINE-1 can serve as a surrogate marker for global DNA methylation. MDA-MB-231 cells exposed to cortisol resulted in a significant decrease (p = 0.05) in the percentage of methylated cytosine found in LINE-1 in comparison to un-treated cells (Fig. 1C). BT-549 cells exposed to cortisol demonstrated a decrease in the percentage of methylated cytosine on LINE-1 but this decrease was not significant. However, a higher percentage of methylated cytosine was detected in MCF-7 cells treated with cortisol in comparison to untreated cells but this was not significant (Fig. 1C).

### 3.2. Loss of methylation on promoter region of ESR1 and increase in ER-α in response to cortisol treatment in TNBC

**Fig. 2.**
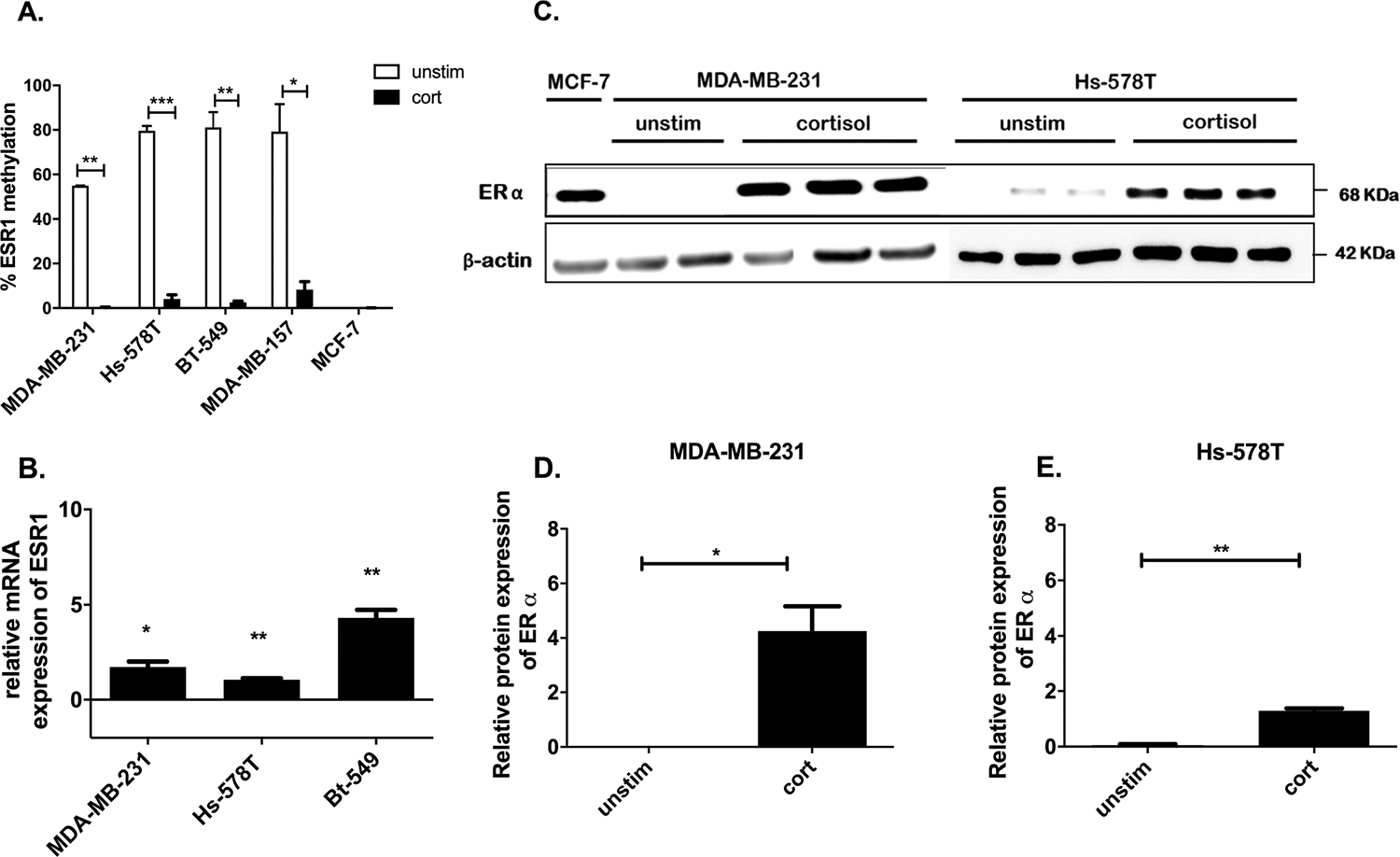
Loss of methylation on promoter region of ESR1 and increase in ER-α in response to cortisol treatment in TNBC. A. qRT-PCR results demonstrating loss of methylation of the promoter region of ESR1 gene in TNBC cells and no change in MCF-7 cells in response to cortisol treatment. B. qRT-PCR results demonstrating the increase in ESR1 gene expression in response to cortisol treatment in TNBC cells. C. Western blot of MDA-MB-231 and Hs-578T was performed using antibodies against ER and βActin (βActin was used as endogenous control of expression). MCF-7 was used as a positive control for ER expression. D & E Represent quantification of ERα expression from corresponding western blot performed using imagej software. * Represents significant difference (* p < 0.05, ** p < 0.01, ***p < 0.001).

As the tumour suppressor array analysis showed a decrease in methylation of *ESR1* promoter region in response to 20 days of cortisol treatment, we further investigated that loss of methylation of *ESR1* in TNBC cells. MDA-MB-231, MDA-MB-157, Hs-578T, BT-549 and ER^+^ MCF-7 cells (served as a control) were treated with 5 μM cortisol for 20 days. Treatment with cortisol led to a significantly decrease in methylation on the promoter region of ESR1 in MDA-MB-231 (p = 0.0027), Hs 578T (p = 0.0001), BT-549 (p = 0.0016), and MDA-MB-157 (p = 0.0313) cells. MCF-7 cells showed no change in ESR1 methylation in response to cortisol treatment compared to untreated cells (Fig. 2A). To study whether the loss of methylation of *ESR1* found in TNBC in response to cortisol treatment is sufficient to up-regulate the ESR1 mRNA expression, MDA-MB-231, Hs-578T and BT-549 cells were treated with 5 μM of cortisol for 20 days. *ESR1* expression was quantified using the ΔΔCt method. There was a significant increase in *ESR1* in all 3 TNBC cells in response to cortisol treatment (Fig. 2B). We next investigated whether the loss of methylation was translated into ER protein expression in TNBC cells by western blot analysis. Cortisol exposed cells showed the re-expression of ER protein in both TNBC cells (Fig. 2C–E).

### 3.3. Cortisol restores sensitivity to Fulvestrant in TNBC cells.

**Fig. 3.**
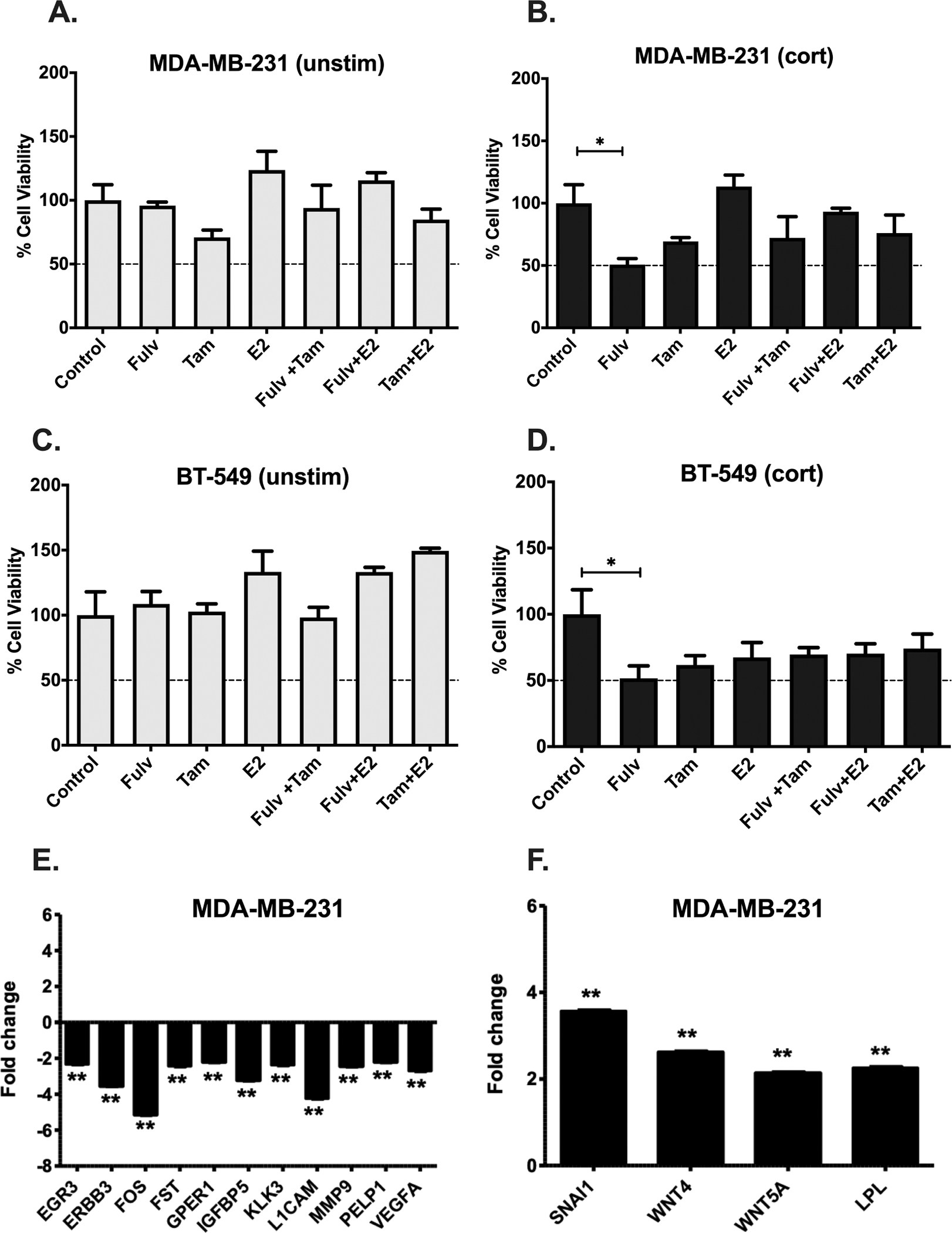
MTT demonstrating sensitivity of TNBC to Tamoxifen and Fulvestrant after 20 days of cortisol treatment and gene expression analysis of oestrogen receptor signalling genes using RT^2^ PCR profiler. A and B: Cell viability data of control (unstim) MDA-MB-231 cells (A) MDA-MB-231 cells treated with cortisol (cort) (B), control (unstim) BT-459 cells (C) BT-549 cells treated with cortisol (cort) (D) E, and F: qRT-PCR results analysis of RT^2^ PCR profiler of oestrogen receptor signalling genes. Results are presented as fold change calculated using the ΔΔCt method normalised to control cells (un-treated). * Represents significant difference (* p < 0.05, ** p < 0.01).

To test the function of the re-expressed ER in TNBC, we examined if cortisol exposure in TNBC with re-expressed ER protein could respond to two ER antagonists. MDA-MB-231 and BT-549 cells were treated with/without 5 μM of cortisol for 20 days prior to Tam, Fulv and E2. Cells were then were incubated with either 1 μM of Fulvestrant (Fulv) and/or 1 μM of Tamoxifen (Tam) for 7 days. Cells were also treated with Estradiol (E2) at a concentration of 10 nM to test the response of cells to an ER agonist. The concentrations of treatments used were optimised by treating MCF-7 (ER^+^) cells with different doses of Tam, Fulv, and E2 (Supplementary Fig. 1). Cell viability was assessed by MTT. The viability of cortisol-treated MDA-MB-231, and BT-549 cells was significantly decreased in response to Fulv. This suggests that the re-expressed ER previously described is actually active, functional and facilitates a response to Fulvestrant (Fig. 3A–D).

To determine if cortisol could alter downstream ER target genes, mRNA expression of 84 different genes was analysed using RT^2^ Profiler PCR Array for Human Estrogen Receptor Signalling genes. MDA-MB-231 cells treated with cortisol demonstrated a downregulation of more than 2-fold in *EGR3, ERBB3, FOS, FST, GPER1, IGFBR3, KLK3, L1CAM, MMP9, PELP1,* and *VEGFA* (Fig. 3E), and an increase of more than 2-fold in *SNAI1, WNT4, WNT5A,* and *LPL* (Fig. 3F).

### 3.4. Glucocorticoid-induced changes in DNMT expression in breast cancer cell lines:

**Fig. 4.**
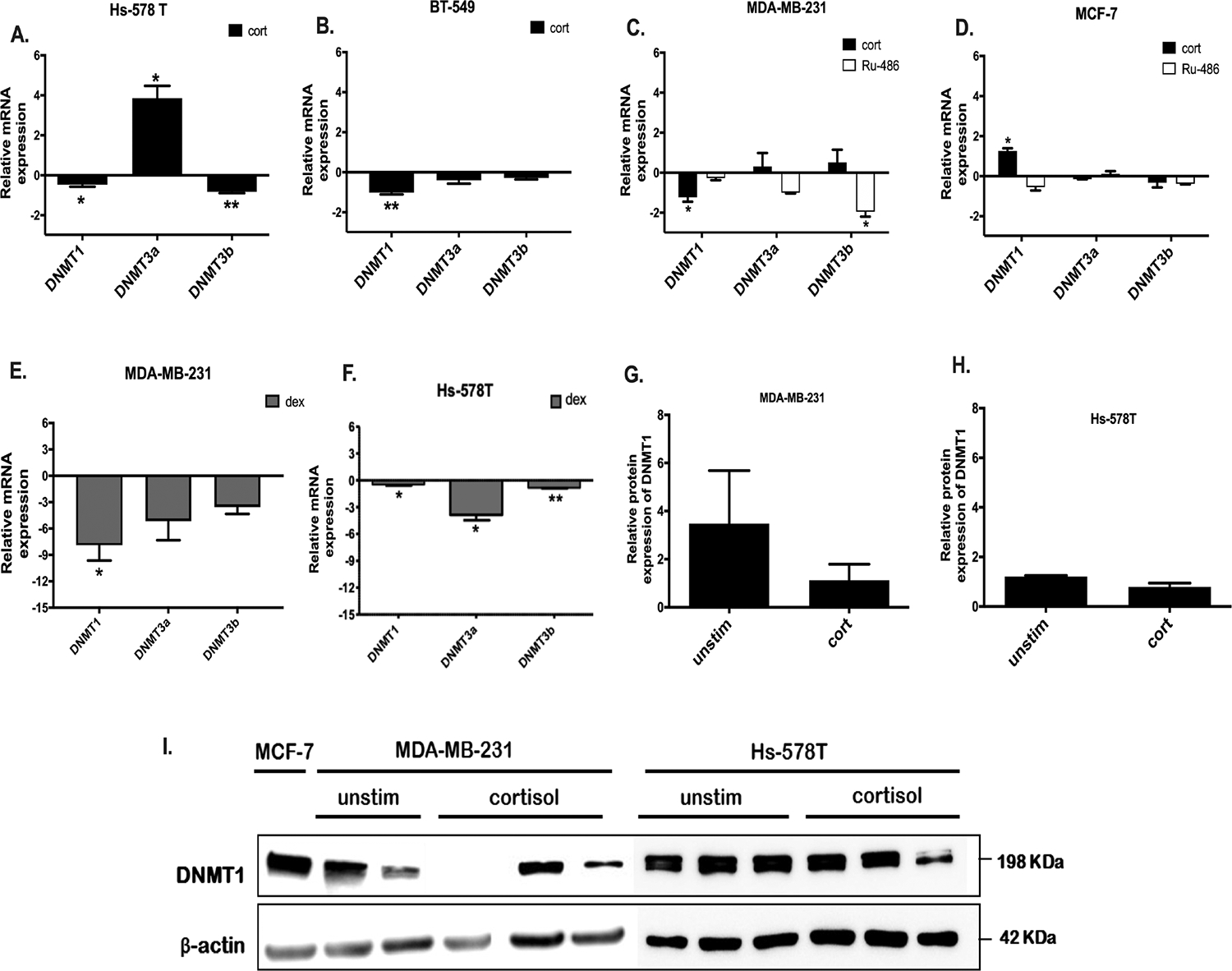
Changes in expressions of DNMTs in response to GC’s. A-B qRT-PCR of DNMT expression of Hs-578T cells (A) BT-549 (B) treated with cort. C-D qRT-PCR of DNMTs expression of MDA-MB-231 cells (C) and MCF-7 cells (D) exposed to cort, with and without RU-486. E-F. qRT-PCR of DNMTs expression of MDA-MB-231 cells (E) and HS-578T (F) treated with Dex for 24 hrs F. β-Actin was used as an endogenous control. Results are presented as relative quantification calculated using the ΔΔCt method normalised to control cells (untreated). Mean ± SEM expressed, and one sample *t*-test was used to compare the mean significance to a hypothetical value of 0 (untreated cells). I. Western blot of MDA-MB-231 and Hs-578T cells was performed using antibodies against DNMT1 and βActin. G-H Represent quantification of ER α expression from corresponding western blot performed using imagej software. Western blots for DNMT1 was performed at the same time as western blot presented in Fig. 2C * Represents significant difference (* p < 0.05, ** p < 0.01).

To investigate other epigenetic effects of cortisol exposure on breast cancer, breast cancer cell lines were treated daily with cortisol (5 μM) for 20 days. The expression of *DNMTs* were evaluated in MCF7 cells (ER^+^) and Hs-578T, BT-549, and MDA-MB-231 cells (TNBC) using qRT-PCR. Results are expressed as relative quantification to untreated cells. Hs 578T, BT-549 and MDA-MB-231 cells exposed to cortisol resulted in a significant decrease in *DNMT1* (p = 0.0429, p = 0.0051, p = 0.0307). Furthermore HS-578T cells treated with cortisol resulted in a significant decrease in *DNMT3b* (p = 0.0060); whereas *DNMT3a* was significantly increased in response to cortisol (p = 0.0244). *DNMT3a* and *b* were unchanged in both BT-549 and MDA-MB-231 cells (Fig. 4A, B, and C). However, MCF-7 cells exposed to cortisol resulted in a significant increase of *DNMT1* (p = 0.0355) and *DNMT3a, and DNMT3b* were not changed (Fig. 4D). In summary, cortisol exposure for 20 days to TNBC caused a significant decrease of *DNMT1* expression, whereas in ER^+^ MCF-7 cells, resulted in a significant increase of *DNMT1*.

To block GR, cells from both MDA-MB-231 and MCF-7 cells were treated with the GR antagonist, mifepristone (RU-486). RU-486 was added at a concentration of 1 μM daily, 30 min prior to the addition of 5 μM cortisol for 20 days and *DNMTs* were examined by qRT-PCR In MDA-MB-231 cells, the cortisol-induced decrease in *DNMT1* was blocked by RU-486; as was the cortisol-induced increase in *DNMT1* expression in MCF-7 cells (Fig. 4C, and D respectively). To fully determine if these cortisol effects on *DNMTs* were mediated by the GR, an ER^+^ T-47D cells (low GR expressing cells) (Reeder et al., 2015) were treated with 5 μM cortisol every 24 hrs for 20 days. Cortisol had no significant effects on *DNMT1, DNMT3a and DNMT3b* expression compared to controls (Supplementary Fig. 2).

To evaluate if the changes in *DNMTs* expressions can be altered using other GCs, breast cancer cell lines were treated with Dexamethasone (Dex) a synthetic GC that is more potent that cortisol (Nebesio, 2016). MDA-MB-231, and Hs-578T cells were treated with 1 μM of Dex for 24 hrs. The expression of *DNMT1* in MDA-MB-231 cells was significantly decreased in response to 24 hrs of Dex treatment (p = 0.0458), while the expression of *DNMT3a*, and *DNMT3b* were not changed which matches what we observed with cort (Fig. 1E). In Hs-578T cells, the expression of *DNMT1, DNMT3a* and *DNMT3b* were found to be significantly decreased after 24 hrs of exposure to Dex (p = 0.0429, 0.0244, 0.0060 respectively) (Fig. 4F). The GR antagonist (RU-486) abrogated the Dex decrease in *DNMT1* (Supplementary Fig. 3).

We next analysed the effect of cortisol on DNMT1 protein using western blot in MDA-MB-231 and Hs-578T cells. However, there were no significant changes in DNMT1 protein expression in response to cortisol treatment (Fig. 4G, H, and I).

### 3.5. Correlation of psychological stress and decrease in methylation levels of ER and increase in ESR1 gene expression in an MDA-MB-231 xenograft mouse model

**Fig. 5.**
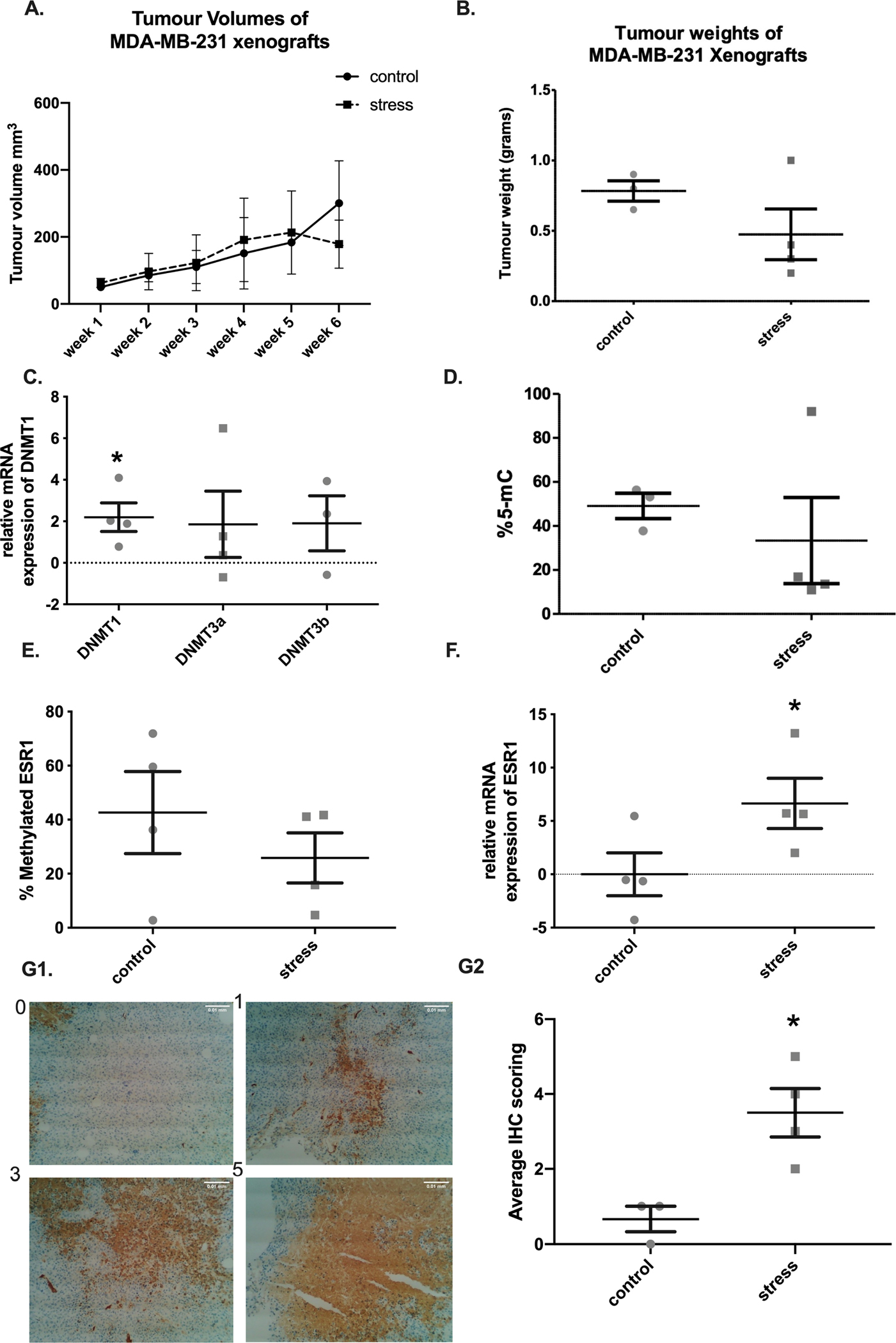
Correlation of psychological stress and decrease in methylation levels of ER and increase in *ESR1* expression in an MDA-MB-231 xenograft mouse model. A. Tumour volumes in non-stressed (control n = 4) and stressed (stress = 4) mice. B. Tumour weights in non-stressed (control) and stressed (stress) group. C. qRT-PCR analysis of *DNMT1*, *DNMT3a*, and *DNMT3b* mRNA expression in tissues of MDA-MB-231 xenografts. D. The percentage of methylated cytosine correlated with detectable CpG residues in LINE-1 between non-stressed (control) and stressed (RS) primary tumour DNA of MDA-MB-231 xenografts using ELISA. E. qRT-PCR results demonstrating the methylation change on the promoter region of *ESR1* in primary tumours of non-stressed (control) and stressed MDA-MB-231 derived xenografts. F. qRT-PCR mRNA expression analysis of ESR1 in the primary tumours of non-stressed (control) and stressed MDA-MB-231 derived xenografts. Results are presented as relative quantification calculated using the ΔΔCt method normalised to control group Mean ± SEM expressed and one sample *t*-test was used to compare the mean significance to a hypothetical value of 0G1 shows a representation of proportion scoring of ER staining from 0 to 5, where 0 = no cells are stained, 1=<1% of cells are stained, 3 = 11–33% of cells are stained, and 5 = 67–100% of cells are stained. Scale bar is 0.01 mm. G2. IHC analysis of ER expression in paraffin embedded sections of tumours from stressed MDA-MB-231 xenograft mice compared to non-stressed (control), a *t*-test was used to compare the mean significance between the two groups (*p < 0.05).

To assess the effects of psychological stress on the expression of DNMTs, a restraint stress MDA-MB-231 xenograft model was developed. Tumour volumes were monitored from injection day to the end point of the study, and tumours’ weights were measured at necropsy. There were no significant differences in tumour volumes or weight of the tumour between the control and the restraint stress groups (Fig. 5A–B). RNA was extracted from harvested tumours; cDNA was synthesised and the expression of the DNMTs was analysed using qRT-PCR. Results were normalised to control group (non-stress group). Surprisingly, there was a significant increase in *DNMT1* in tumours isolated from RS mice but no significant difference in DNMT3a, and DNMT3b in comparison to the non-stressed group (Fig. 5C).

The effects of psychological stress on global DNA methylation were then evaluated by assessing the percentage of methylated cytosine on LINE-1. Although the difference in means of the methylated cytosine between the control and RS group was not statistically significant, the data indicate that the percentage of methylated cytosine on LINE-1 in the majority of mice in the stress group is less compared to the control group (Fig. 5D).

To assess the effect of psychological stress on the methylation pattern of ESR1, qRT-PCR was used to evaluate the methylation level on the promoter region of *ESR1* in DNA extracted from tumour tissues of the xenografts. There was no significant decrease in the average methylation percentage in stress group compared to the control group. However, there was a trend indicating that restraint stress may exert an effect on the methylation status of ESR1 in the stress group (Fig. 5E). To investigate whether the loss of methylation on the promoter region of ESR1 was sufficient to upregulate the expression of ESR1, total RNA was extracted from the same tumours and mRNA expression of ESR1 was assessed using qRT-PCR. *ESR1* mRNA expression was significantly higher in the stressed group compared to control group (p = 0.0451) (Fig. 5F). Immunohistochemical assessment (IHC) was used to determine the ER protein levels in tumours harvested from stress group and control group MDA-MB-231 xenografts. Paraffin-embedded sections were incubated with a primary antibody against ER and were blindly scored from 0 to 5 (Figure 5 G1). (Ilíc et al., 2019). There was a significant increase in ER in tumours form the RS group when compared to the control group (p = 0.0286) (Figure 5 G2).

## 4. Discussion

We have shown that cortisol exposure to TNBC cells exhibited changes of methylation levels on promoter regions of tumour suppressor genes, and changes of global methylation. These changes suggest that cortisol leads to epigenetic modification through the alteration of gene expression of key epigenetic markers. Furthermore, our findings also demonstrated that long term exposure of cortisol caused a significant loss of methylation on ESR1 gene, a significant increase in *ESR1* mRNA, and an upregulation of ERα protein. Cell viability was also significantly reduced in response to endocrine therapy treatment, implying the restoration of sensitivity to endocrine treatment. Although cells were grown in charcoal stripped FBS and phenol red-free media, it is possible that the media used might have traces of hormone content (Gardiner-Garden and Frommer, 1987).

The downstream effect of cortisol on ER target genes was also explored. Cortisol regulates ER response genes, resulting in a less aggressive phenotype. For example, the upregulation of Proline-, glutamic acid-and leucine-rich protein1 (*PELP1*), an oestrogen receptor coactivator and proto-oncogene, has been reported to be associated with poor prognosis in breast cancer patients (Habashy et al., 2010), and with resistance to endocrine therapy *in vitro* (Flågeng et al., 2015). However, cortisol downregulated the expression of *PELP1* suggesting a favourable phenotype. The over expression of ErB-B2 receptor tyrosine kinase 3 (*ERBB3*) has been associated with malignant phenotypes as it plays a crucial role in cell proliferation and migration (Wang, 2017; Mujoo et al., 2014). Our data suggest that cortisol led to the downregulation of *ERBB3*. Our results are consistent with a study that demonstrated the degradation of *ERBB3* in response to E2 treatment in ER^+^ MCF-7 cells (Suga et al., 2018). The overexpression of the cell adhesion molecule *(L1CAM*) has been correlated with tumour aggressiveness and poor prognosis in patients and promotion of breast cancer motility *in vitro* (Kiefel et al., 2012; Zhang, 2015). L1CAM was downregulated in response to cortisol, suggesting a favourable phenotype and less aggressiveness. This suggestion can be also supported by a study that demonstrated the over-expression of L1CAM promoted invasion and migration of TNBC cells, and *L1CAM* was therefore regarded as a driver of tumour progression (Doberstein, 2014). Another study found *L1CAM* to be overexpressed in Fulvestrant resistant MCF-7 cells showing a negative correlation between L1CAM and ER status (Hiscox et al., 2009). The inhibition of the Snail Family transcriptional repressor1 (*SNAI1*) was reported in literature to restore sensitivity to tamoxifen in oestrogen- hyposensitive MCF-7 cells (Scherbakov et al., 2012). This explains our findings showing that cortisol treated TNBC MDA-MB-231 cells demonstrated a partial and non-significant response to tamoxifen, and PCR arrays demonstrated an upregulation of *SNAI1* in cortisol treated MDA-MB-231 cells. This is in line with results of a study demonstrating the downregulation of several genes related to EMT including *SNAI1* upon the co-activation of ER and GR in ER^+^ BC (West et al., 2016). However, other studies have reported a higher expression of *SNAI1* in patients with complete pathological response to therapy, postulating a protective and anti-tumorigenic role for *SNAI1* in breast cancer (Al-Zeheimi, 2019). Another study demonstrated that the regulation of miR-204, -200c, -34a, and -10 plays a role in increasing the survival rate of invasive breast cancer by up-regulating *SNAI1* and other genes (Rahimi et al., 2019). Thus, the demonstrated increase in *SNAI1* expression in response to cortisol treatment could be protective. We also found that *WNT4* was increased in cortisol treated cells. Others have shown that an induced overexpression of *WNT5A in vitro* led to tumour suppressive responses characterised by impaired migration and invasion (Rahimi et al., 2019). Investigators have also correlated reduced expression of *WNT5A* to loss of ER and early relapse in invasive ductal carcinoma patients (Jönsson, 2002; Zhong et al., 2016). Taken together, it is possible that cort can alter the epigenetic landscape of an aggressive breast tumour, to potentially a tumour that is sensitive to endocrine therapy. This epigenetic alteration was achieved without the use of an epigenetic inhibitor, suggesting the role of GC’s in epigenetic modifications.

Interestingly, although we saw a consistent down regulation of DNMT1 in all TNBC cells exposed to both GC’s, whereas the effects on DNMT3a and 3b were variable between cell lines. Differences in DNMT3a and DNMT3b could be due to the differences in characteristics of cells. For example, these cells have different levels of GR and hence, would respond differently to GC treatment. Although initially, the re-expression of the functional ER was hypothesised to be attributed to the down regulation of DNMT1, cortisol did not down regulate DNMT1 protein in TNBC cells and we did not see a down regulation in our xenograft model. However, it is possible that DNMT1 activity is inhibited by cortisol in TNBC cells, and that change in enzymatic activity may be attributed to the re-expression of ER or indeed ER expression may be independent of DNMT1. The response to psychological stress in mice has also led to the loss of methylation of the promoter region of ESR1. Not all mice lost the methylation of the promoter region of ESR1; however, this could be due to the resilience of some animals to stress in comparison to other mice in the study. The loss of methylation found in mice was negatively correlated with the mRNA expression of ESR1. Although it is possible that the epigenetic change could be due to the alteration of DNMT1 expression and/or activity, the detailed mechanism is yet to be investigated.

We have demonstrated that exposure to Gcs re-expresses active ER in TNBC and restores sensitivity to endocrine therapy. The addition of oestrogen did not increase the cell viability however we are not expecting these cells to behave exactly like a classic ER^+^ cell line such as MCF-7. However, evidence in MCF-7 cells also suggests that the E2 responsiveness of oestrogen receptor positive breast cancer cell lines is dependent on an autocrine signal activating the IGF-I receptor. It has also been reported that the activation of GR decreases the stimulatory effect of IGF-1 receptor in different cell lines. Therefore, it is possible that the MDA-MB-231 with re-activated oestrogen receptor do not respond to E2 in the presence of glucocorticoids due the IGF-RI-pathway blockage. The cells still significantly respond to Fulvestrant treatment.

The limitations of our study are that our data was conducted in cell lines and *invivo* mouse model of one TNBC cell line. Further work is ongoing in our laboratory to understand the underlying mechanisms and to establish the effect of tamoxifen and fluvestrant *in vivo*; however, the phenotypic observations made in this paper highlight the very important findings that stress hormones can induce a loss of ER-α gene methylation and restoration of sensitivity to Fulvestrant in TNBC.

Clinical studies suggest receptor conversion of ERα from negative to positive in the course of disease progression in distant breast cancer metastases (Schrijver et al., 2018; Aurilio et al., 2013; Chang et al., 2011; Hoefnagel, 2013; Bernsdorf, 2011). Although these studies attribute the conversion mainly to the heterogeneity of the disease, these studies do not assess the stress of patients nor the GR status. The change in ER expression in our study could potentially contribute to the debatable explanations about this phenomenon and support the encouragement of re-assessing the hormonal status of tumours and metastases during the progression of the disease (Schrijver et al., 2018; Bernsdorf, 2011). Our results suggest that psychological stress can lead to the re-expression of ER altering the phenotype of breast cancer. This could affect the course of treatment for patients, as tumours expressing ER can be targeted using different ER antagonists and are more responsive to endocrine therapy. In summary, although there was a diminished enthusiasm for re-expressing ER in TNBC in the field (Yan, 2003; Yang, 2001; Sabnis, 2011; Munzone, 2006; Fan, 2008), our findings suggest that restoring the ER expression along with its sensitivity to endocrine therapy in stressed patients is worth pursuing.

## Supplementary Material

Supp Data

## Data Availability

Data will be made available on request.

## Abbreviations:

ADAM23: ADAM metallopeptidase domain 23
AHR: Aryl hydrocarbon receptor
AKAP1: A kinase (PRKA) anchor protein 1
APBB1: Amyloid beta (A4) precursor protein-binding, family B, member 1 (Fe65)
APC: Adenomatous polyposis coli
AR: Adrenergic receptor
ATM: Ataxia telangiectasia mutated
BCAR1: Breast cancer anti-estrogen resistance 1
BCL2L1: BCL2-like 1
BDNF: Brain-derived neurotrophic factor
BIRC5: Baculoviral IAP repeat containing 5
BMP4: Bone morphogenetic protein 4
BMP6: Bone morphogenetic protein 6
BMP7: Bone morphogenetic protein 7
BRCA1: Breast cancer 1, early onset
BRCA1: Breast cancer 1
BRCA2: Breast cancer 2, early onset
C3: Complement component 3
CADM1: Cell adhesion molecule 1
CALCA: Calcitonin-related polypeptide alpha
CAV1: Caveolin 1, caveolae protein, 22kDa
CAV1: Caveolin 1, caveolae protein
CCL2: Chemokine (C-C motif) ligand 2
CCNA1: Cyclin A1
CCND1: Cyclin D1
CCND2: Cyclin D2
CDH1: Cadherin 1, type 1, E-cadherin
CDH13: Cadherin 13, H-cadherin (heart)
CDKN1B: Cyclin-dependent kinase inhibitor 1B (p27)
CDKN1C: Cyclin-dependent kinase inhibitor 1C (p57)
CDKN2A: Cyclin-dependent kinase inhibitor 2A (p16)
CDKN2B: Cyclin-dependent kinase inhibitor 2B (p15)
CDX2: Caudal type homeobox 2
CHFR: Checkpoint with forkhead and ring finger domains
CITED2: Cbp/p300-interacting transactivator, with Glu/Asp-rich carboxy-terminal domain, 2
CKB: Creatine kinase, brain
CLSTN1: Calsyntenin 1
CORT: Cortisol
CST6: Cystatin E/M
CTGF: Connective tissue growth factor
CTSD: Cathepsin D
CTSZ: Cathepsin Z
CXCL12: Chemokine (C-X-C motif) ligand 12
CYP19A1: Cytochrome P450, family 19, subfamily A, polypeptide 1
CYP1A1: Cytochrome P450, family 1, subfamily A, polypeptide 1
CYP1B1: Cytochrome P450, family 1, subfamily B, polypeptide 1
DAPK1: Death-associated protein kinase 1
DEX: Dexamethasone
DNMT: DNA methyl transferase
DSC3: Desmocollin 3
E2: Estradiol
EBAG9: Estrogen receptor binding site associated, antigen, 9
EFNA5: Ephrin-A5
EGR3: Early growth response 3
EPB41L3: Erythrocyte membrane protein band 4.1-like 3
EPCAM: Epithelial cell adhesion molecule
ER: Oestrogen Receptor
ERBB2: V-erb-b2 erythroblastic leukemia viral oncogene homolog 2, neuro/glioblastoma derived oncogene homolog (avian)
ERBB3: V-erb-b2 erythroblastic leukemia viral oncogene homolog 3 (avian)
ESR1: Estrogen receptor 1
ESR2: Estrogen receptor 2 (ER beta)
FHIT: Fragile histidine triad gene
FOS: FBJ murine osteosarcoma viral oncogene homolog
FOXA1: Forkhead box A1
FST: Follistatin
Fulv: Fulvestrant
G6PD: Glucose-6-phosphate dehydrogenase
GADD45A: Growth arrest and DNA-damage-inducible, alpha
GC: Glucocorticoid
GPC3: Glypican 3
GPER1: G protein-coupled estrogen receptor 1
GR: Glucocorticoid receptor
GSTP1: Glutathione S-transferase pi 1
HER-2: Human epidermal growth factor receptor 2
HIC1: Hypermethylated in cancer 1
HOXA5: Homeobox A5
HSP90AA1: Heat shock protein 90kDa alpha (cytosolic), class A member 1
IGF1: Insulin-like growth factor 1 (somatomedin C)
IGFBP4: Insulin-like growth factor binding protein 4
IGFBP5: Insulin-like growth factor binding protein 5
IRS1: Insulin receptor substrate 1
JUNB: Jun B proto-oncogene
KLK3: Kallikrein-related peptidase 3
L1CAM: L1 cell adhesion molecule
LGALS1: Lectin, galactoside-binding, soluble, 1
LPL: Lipoprotein lipase
LTBP1: Latent transforming growth factor beta binding protein 1
MGMT: O-6-methylguanine-DNA methyltransferase
MTT: Microculture Tetrazolium
NS: Non-stressed
PCR: Polymerase chain reaction
PR: Progesterone Receptor
PRDM2: PR domain containing 2, with ZNF domain
PTEN: Phosphatase and tensin homolog
PTGS2: Prostaglandin-endoperoxide synthase 2 (prostaglandin G/H synthase and cyclooxygenase)
PYCARD: PYD and CARD domain containing
RASSF1: Ras association (RalGDS/AF-6) domain family member 1
RS: Restraint stress
SFN: Stratifin
SLIT2: Slit homolog 2 (Drosophila)
Tam: Tamoxifen
THBS1: Thrombospondin 1
TNBC: Triple negative breast cancer
TNFRSF10C: Tumor necrosis factor receptor superfamily, member 10c
TP73: Tumor protein p73
