# Supplementary material for: Glucocorticoid induced loss of oestrogen receptor alpha gene methylation and restoration of sensitivity to fulvestrant in triple negative breast cancer
Source: Gene. Author manuscript; Available in PMC 2024 Jun 20. (PMC11188041; doi:10.1016/j.gene.2022.147022)
Supplement: Supp Data [file NIHMS1999351-supplement-Supp_Data.docx]

Supplementary table 1List of genes from RT^2^ Profiler ER Receptor Signalling array

| **Gene Symbol** | **Description** | **Gene Symbol** | **Description** |
| --- | --- | --- | --- |
| **AHR** | Aryl hydrocarbon receptor | MAFF | V-maf musculoaponeurotic fibrosarcoma oncogene homolog F (avian) |
| **AKAP1** | A kinase (PRKA) anchor protein 1 | MED1 | Mediator complex subunit 1 |
| **APBB1** | Amyloid beta (A4) precursor protein-binding, family B, member 1 (Fe65) | MMP9 | Matrix metallopeptidase 9 (gelatinase B, 92kDa gelatinase, 92kDa type IV collagenase) |
| **BCAR1** | Breast cancer anti-estrogen resistance 1 | MTA1 | Metastasis associated 1 |
| **BCL2L1** | BCL2-like 1 | MYC | V-myc myelocytomatosis viral oncogene homolog (avian) |
| **BDNF** | Brain-derived neurotrophic factor | NAB2 | NGFI-A binding protein 2 (EGR1 binding protein 2) |
| **BMP4** | Bone morphogenetic protein 4 | NCOA1 | Nuclear receptor coactivator 1 |
| **BMP7** | Bone morphogenetic protein 7 | NCOA2 | Nuclear receptor coactivator 2 |
| **BRCA1** | Breast cancer 1, early onset | NCOA3 | Nuclear receptor coactivator 3 |
| **C3** | Complement component 3 | NCOR1 | Nuclear receptor corepressor 1 |
| **CAV1** | Caveolin 1, caveolae protein, 22kDa | NCOR2 | Nuclear receptor corepressor 2 |
| **CCL2** | Chemokine (C-C motif) ligand 2 | NOV | Nephroblastoma overexpressed gene |
| **CCND1** | Cyclin D1 | NR0B1 | Nuclear receptor subfamily 0, group B, member 1 |
| **CITED2** | Cbp/p300-interacting transactivator, with Glu/Asp-rich carboxy-terminal domain, 2 | NR0B2 | Nuclear receptor subfamily 0, group B, member 2 |
| **CKB** | Creatine kinase, brain | NR2F6 | Nuclear receptor subfamily 2, group F, member 6 |
| **CTGF** | Connective tissue growth factor | NR3C1 | Nuclear receptor subfamily 3, group C, member 1 (glucocorticoid receptor) |
| **CTSD** | Cathepsin D | NR5A2 | Nuclear receptor subfamily 5, group A, member 2 |
| **CYP19A1** | Cytochrome P450, family 19, subfamily A, polypeptide 1 | NRIP1 | Nuclear receptor interacting protein 1 |
| **CYP1A1** | Cytochrome P450, family 1, subfamily A, polypeptide 1 | NRP1 | Neuropilin 1 |
| **EBAG9** | Estrogen receptor binding site associated, antigen, 9 | PDZK1 | PDZ domain containing 1 |
| **EFNA5** | Ephrin-A5 | PELP1 | Proline, glutamate and leucine rich protein 1 |
| **EGR3** | Early growth response 3 | PGR | Progesterone receptor |
| **ERBB2** | V-erb-b2 erythroblastic leukemia viral oncogene homolog 2, neuro/glioblastoma derived oncogene homolog (avian) | PHB2 | Prohibitin 2 |
| **ERBB3** | V-erb-b2 erythroblastic leukemia viral oncogene homolog 3 (avian) | PTCH1 | Patched 1 |
| **ESR1** | Estrogen receptor 1 | PTGS2 | Prostaglandin-endoperoxide synthase 2 (prostaglandin G/H synthase and cyclooxygenase) |
| **ESR2** | Estrogen receptor 2 (ER beta) | RALA | V-ral simian leukemia viral oncogene homolog A (ras related) |
| **FOS** | FBJ murine osteosarcoma viral oncogene homolog | RARA | Retinoic acid receptor, alpha |
| **FOXA1** | Forkhead box A1 | S100A6 | S100 calcium binding protein A6 |
| **FST** | Follistatin | SAFB | Scaffold attachment factor B |
| **G6PD** | Glucose-6-phosphate dehydrogenase | SNAI1 | Snail homolog 1 (Drosophila) |
| **GPER1** | G protein-coupled estrogen receptor 1 | SOCS3 | Suppressor of cytokine signaling 3 |
| **HSP90AA1** | Heat shock protein 90kDa alpha (cytosolic), class A member 1 | SPP1 | Secreted phosphoprotein 1 |
| **IGF1** | Insulin-like growth factor 1 (somatomedin C) | TFF1 | Trefoil factor 1 |
| **IGFBP4** | Insulin-like growth factor binding protein 4 | TGFA | Transforming growth factor, alpha |
| **IGFBP5** | Insulin-like growth factor binding protein 5 | TGFB3 | Transforming growth factor, beta 3 |
| **IRS1** | Insulin receptor substrate 1 | THBS1 | Thrombospondin 1 |
| **JUNB** | Jun B proto-oncogene | VDR | Vitamin D (1,25- dihydroxyvitamin D3) receptor |
| **KLK3** | Kallikrein-related peptidase 3 | VEGFA | Vascular endothelial growth factor A |
| **L1CAM** | L1 cell adhesion molecule | WISP2 | WNT1 inducible signaling pathway protein 2 |
| **LGALS1** | Lectin, galactoside-binding, soluble, 1 | WNT4 | Wingless-type MMTV integration site family, member 4 |
| **LPL** | Lipoprotein lipase | WNT5A | Wingless-type MMTV integration site family, member 5A |
| **LTBP1** | Latent transforming growth factor beta binding protein 1 | XBP1 | X-box binding protein 1 |

Supplementary table 2 List of genes of EpiTect Methyl Tumour Suppressor Genes array

| **Gene Symbol** | **Gene Description** | **Gene Symbol** | **Gene Description** |
| --- | --- | --- | --- |
| **ADAM23** | ADAM metallopeptidase domain 23 | HOXD11 | Homeobox D11 |
| **BRCA1** | Breast cancer 1 | HS3ST2 | Heparan sulfate (glucosamine) 3-O-sulfotransferase 2 |
| **CCNA1** | Cyclin A1 | HS3ST3B1 | Heparan sulfate (glucosamine) 3-O-sulfotransferase 3B1 |
| **CCND2** | Cyclin D2 | HSD17B4 | Hydroxysteroid (17-beta) dehydrogenase 4 |
| **CDH1** | Cadherin 1, type 1, E-cadherin | ID4 | Inhibitor of DNA binding 4, dominant negative helix-loop-helix protein |
| **CDH13** | Cadherin 13, H-cadherin (heart) | IGFBP7 | Insulin-like growth factor binding protein 7 |
| **CDKN1C** | Cyclin-dependent kinase inhibitor 1C (p57) | IGFBPL1 | Insulin-like growth factor binding protein-like 1 |
| **CDKN2A** | Cyclin-dependent kinase inhibitor 2A (p16) | JUP | Junction plakoglobin |
| **ESR1** | Estrogen receptor 1 | KLK10 | Kallikrein-related peptidase 10 |
| **GSTP1** | Glutathione S-transferase pi 1 | LOX | Lysyl oxidase |
| **HIC1** | Hypermethylated in cancer 1 | MEN1 | Multiple endocrine neoplasia I |
| **MGMT** | O-6-methylguanine-DNA methyltransferase | MLH1 | MutL homolog 1, colon cancer, nonpolyposis type 2 |
| **PRDM2** | PR domain containing 2, with ZNF domain | MSX1 | Msh homeobox 1 |
| **PTEN** | Phosphatase and tensin homolog | MUC2 | Mucin 2, oligomeric |
| **PTGS2** | Prostaglandin-endoperoxide synthase 2 (prostaglandin G/H synthase and cyclooxygenase) | MYOD1 | Myogenic differentiation 1 |
| **PYCARD** | PYD and CARD domain containing | PALB2 | Partner and localizer of BRCA2 |
| **RASSF1** | Ras association (RalGDS/AF-6) domain family member 1 | PAX5 | Paired box 5 |
| **SFN** | Stratifin | PDLIM4 | PDZ and LIM domain 4 |
| **SLIT2** | Slit homolog 2 (Drosophila) | PER1 | Period homolog 1 |
| **THBS1** | Thrombospondin 1 | PER2 | Period homolog 2 |
| **TNFRSF10C** | Tumor necrosis factor receptor superfamily, member 10c | PGR | Progesterone receptor |
| **TP73** | Tumor protein p73 | PLAGL1 | Pleiomorphic adenoma gene-like 1 |
| **APC** | Adenomatous polyposis coli | PRKCDBP | Protein kinase C, delta binding protein |
| **ATM** | Ataxia telangiectasia mutated | PROX1 | Prospero homeobox 1 |
| **BIRC5** | Baculoviral IAP repeat containing 5 | RARB | Retinoic acid receptor, beta |
| **BMP6** | Bone morphogenetic protein 6 | RARRES1 | Retinoic acid receptor responder (tazarotene induced) 1 |
| **BRCA2** | Breast cancer 2, early onset | RB1 | Retinoblastoma 1 |
| **CADM1** | Cell adhesion molecule 1 | RBP1 | Retinol binding protein 1, cellular |
| **CALCA** | Calcitonin-related polypeptide alpha | RRAD | Ras-related associated with diabetes |
| **CAV1** | Caveolin 1, caveolae protein | RUNX3 | Runt-related transcription factor 3 |
| **CDKN1B** | Cyclin-dependent kinase inhibitor 1B (p27) | SFRP1 | Secreted frizzled-related protein 1 |
| **CDKN2B** | Cyclin-dependent kinase inhibitor 2B (p15) | SFRP2 | Secreted frizzled-related protein 2 |
| **CDX2** | Caudal type homeobox 2 | SLC5A8 | Solute carrier family 5 (iodide transporter), member 8 |
| **CHFR** | Checkpoint with forkhead and ring finger domains | SLIT3 | Slit homolog 3 |
| **CLSTN1** | Calsyntenin 1 | SYK | Spleen tyrosine kinase |
| **CST6** | Cystatin E/M | TERT | Telomerase reverse transcriptase |
| **CTSZ** | Cathepsin Z | TGFB2 | Transforming growth factor, beta 2 |
| **CXCL12** | Chemokine (C-X-C motif) ligand 12 | TGFBI | Transforming growth factor, beta-induced |
| **CYP1B1** | Cytochrome P450, family 1, subfamily B, polypeptide 1 | TGFBR1 | Transforming growth factor, beta receptor 1 |
| **DAPK1** | Death-associated protein kinase 1 | TIMP3 | TIMP metallopeptidase inhibitor 3 |
| **DSC3** | Desmocollin 3 | TNFRSF10D | Tumor necrosis factor receptor superfamily, member 10d |
| **EPB41L3** | Erythrocyte membrane protein band 4.1-like 3 | TWIST1 | Twist homolog 1 |
| **EPCAM** | Epithelial cell adhesion molecule | VHL | Von Hippel-Lindau tumor suppressor |
| **FHIT** | Fragile histidine triad gene | WIF1 | WNT inhibitory factor 1 |
| **GADD45A** | Growth arrest and DNA-damage-inducible, alpha | WT1 | Wilms tumor 1 |
| **GPC3** | Glypican 3 | WWOX | WW domain containing oxidoreductase |
| **HOXA5** | Homeobox A5 | ZMYND10 | Zinc finger, MYND-type containing 10 |


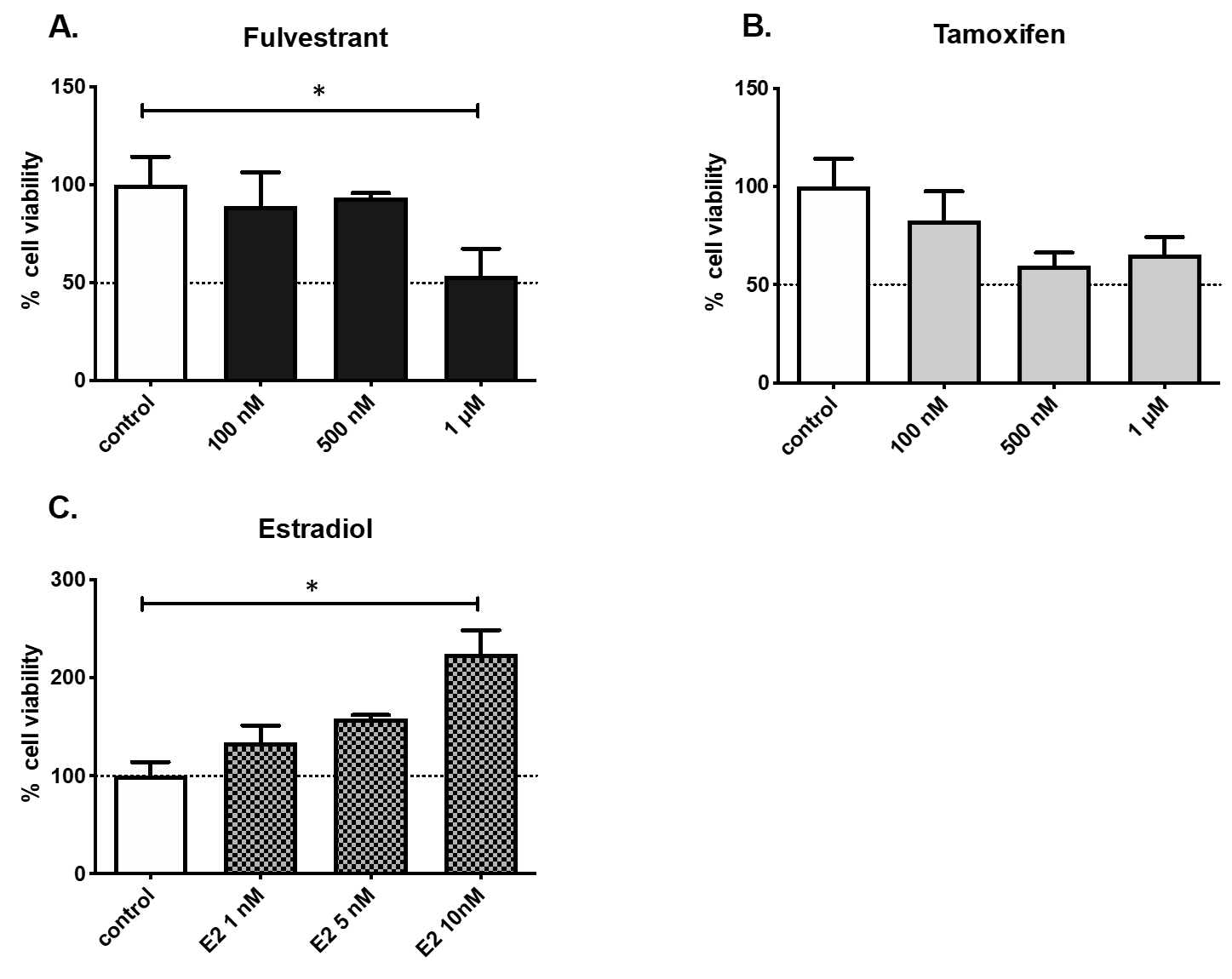


Supplementary Figure 1 MTT of MCF-7 cells treated with different doses of Fulvestrant (A), Tamoxifen (B), and Estradiol (C). MCF-7 cells were plated at a density of 3000/well in 200 μl of DMEM +10% FBS media. Cells were then treated with different doses of Fulvestrant and Tamoxifen (100 nM, 500nM, and 1μM), cells were also treated with different concentrations of Estradiol (E2) (1nM, 5nM, and 10nM). MCF-7 cells were incubated with treatment for 7 days, and an MTT was then preformed to determine cell viability. MTT results also demonstrated a significant increase in viability when treated with 10nM of E2. One-way ANOVA was used to compare between groups, with Bonferroni correction.* represents significant difference (* p<0.05)

Supplementary Figure 2 DNMT1, DNMT3a and DNMT3b mRNA expression in response to cortisol treatment in T47D cells. T47D cells were treated with 5uM of cortisol for 20 days. Total RNA was then extracted, and cDNA was synthesised to evaluate DNMT1, DNMT3a, and DNMT3b mRNA expression using qRT-PCR. β-Actin was used as an endogenous control. Results are presented as relative quantification calculated using the ΔΔCt method normalised to control cells (un-treated). Mean ± SEM expressed and one sample t-test was used to compare the mean significance to a hypothetical value of 0 (untreated cells).

Supplementary Figure 3. The change in *DNMT*1 expression in MDA-MB-231 cell line was abrogated when treated with GR antagonist RU-486. Breast cancer cells from MDA-MB-231 cells were treated with 1µM of dexamethasone (dex) for 24 hrs days with and without RU-486. RU-486 was added 30 min prior to the addition of dexamethasone at a concentration of 1µM. Total RNA was then extracted, and cDNA was synthesised to evaluate *DNMT*1, *DNMT*3a, and *DNMT*3b mRNA expression using qRT-PCR. β-Actin was used as an endogenous control. Results are presented as relative quantification calculated using the ΔΔCt method normalised to control cells (untreated). Mean ± SEM expressed and one sample t-test was used to compare the mean significance to a hypothetical value of 0 (untreated cells).* represents significant difference (* p<0.05)

| Stressed mice | |
| --- | --- |
| x20 | X40 |
| Mouse 1  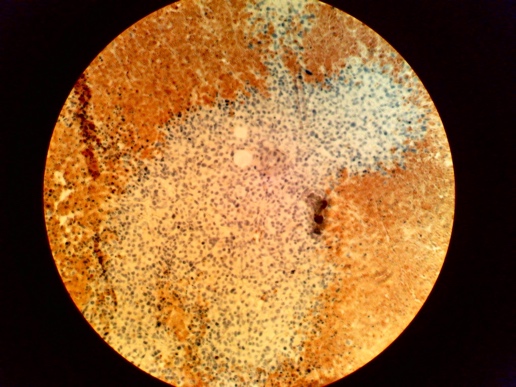 | Mouse 1  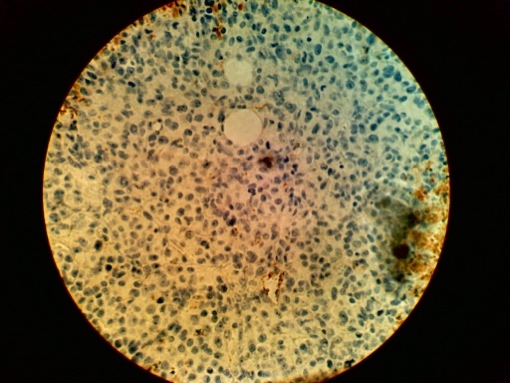 |
| Mouse 2  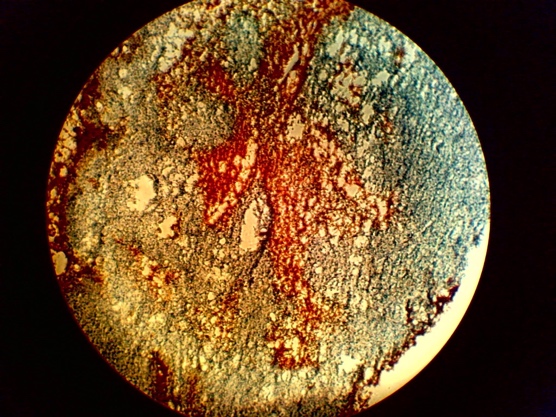 | Mouse 2  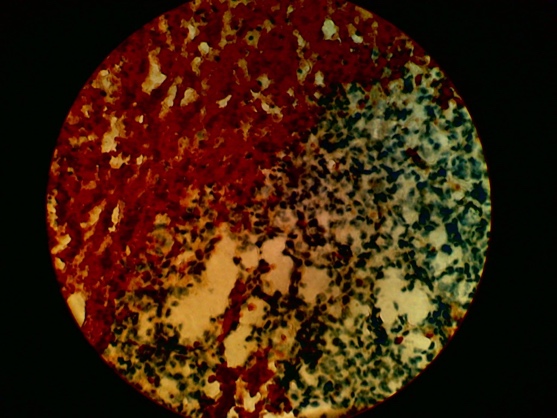 |
| Mouse 3  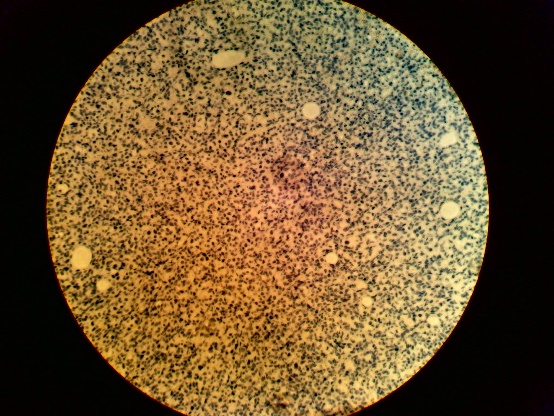 | Mouse 3  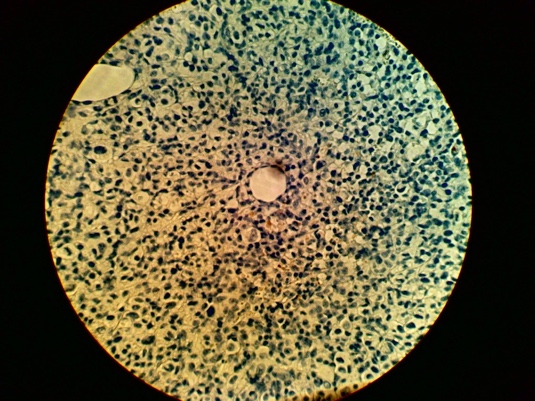 |

Supplementary Figure 4 IHC Images of mammary tumours isolated from stressed mice, magnification x20, and x40. Images shown are representation of proportion scoring of ER staining from 0-5, where 0 = no cells are stained, 1= <1% of cells are stained, 3= 11-33% of cells are stained, and 5= 67-100% of cells are stained

| Non-stressed mice | |
| --- | --- |
| x 20 | x 40 |
| Mouse 1  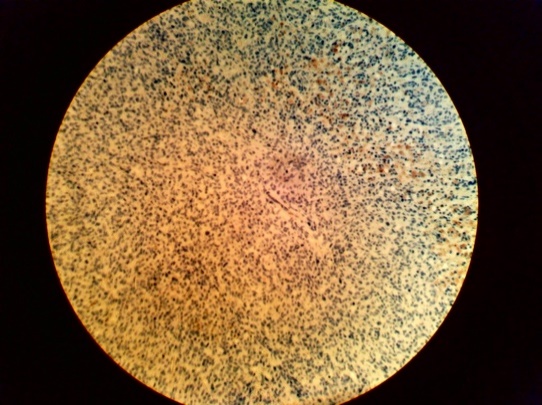 | Mouse 1  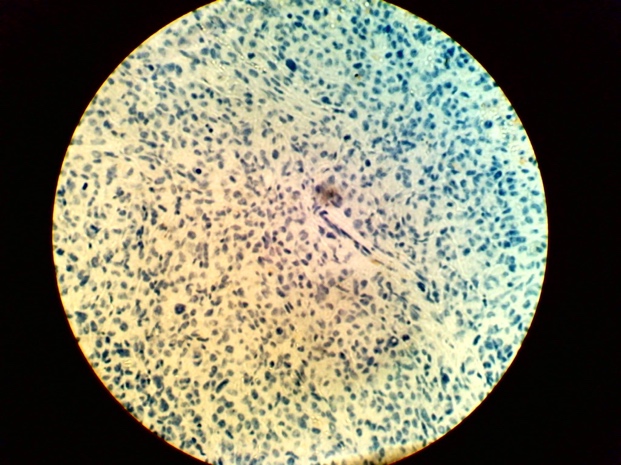 |
| Mouse 2  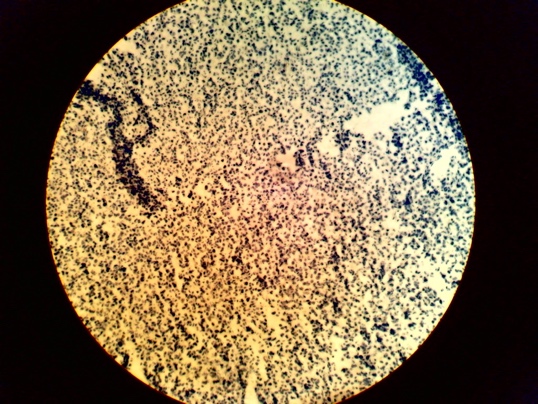 | Mouse 2  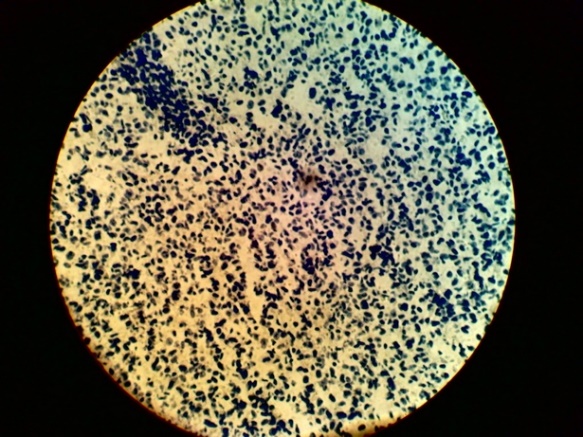 |
| Mouse 3  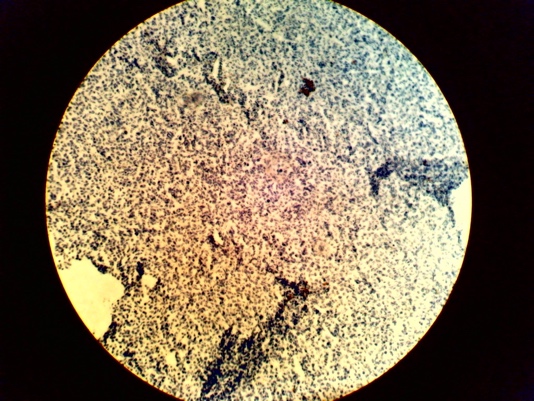 | Mouse3  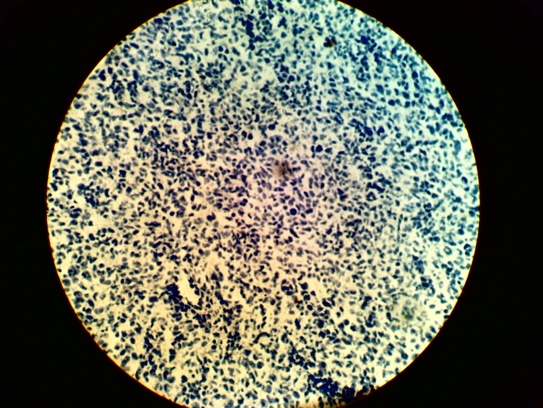 |

Supplementary Figure 5 IHC Images of mammary tumours from non-stressed mice,magnification x20, and x40. Images shown are representation of proportion scoring of ER staining from 0-5, where 0 = no cells are stained, 1= <1% of cells are stained, 3= 11-33% of cells are stained, and 5= 67-100% of cells are stained
